# Statin-induced anti-proliferative effects via cyclin D1 and p27 in a window-of-opportunity breast cancer trial

**DOI:** 10.1186/s12967-015-0486-0

**Published:** 2015-04-29

**Authors:** Maria Feldt, Olöf Bjarnadottir, Siker Kimbung, Karin Jirström, Pär-Ola Bendahl, Srinivas Veerla, Dorthe Grabau, Ingrid Hedenfalk, Signe Borgquist

**Affiliations:** Division of Oncology and Pathology, Department of Clinical Sciences, Lund University, Lund, Sweden; Department of Oncology, Skåne University Hospital, Lund, Sweden

**Keywords:** Cyclin D1, P27, Ki67, Statins, Breast cancer

## Abstract

**Purpose:**

Cholesterol lowering statins have been demonstrated to exert anti-tumoral effects on breast cancer by decreasing proliferation as measured by Ki67. The biological mechanisms behind the anti-proliferative effects remain elusive. The aim of this study was to investigate potential statin-induced effects on the central cell cycle regulators cyclin D1 and p27.

**Experimental design:**

This phase II window-of-opportunity trial (Trial registration: ClinicalTrials.gov NCT00816244, NIH) included 50 patients with primary invasive breast cancer. High-dose atorvastatin (80 mg/day) was prescribed to patients for two weeks prior to surgery. Paired paraffin embedded pre- and post-statin treatment tumor samples were analyzed using immunohistochemistry for the expression of estrogen receptor (ER), progesterone receptor (PR), human epidermal growth factor receptor 2 (HER2), and the cell cycle regulators cyclin D1 and p27. Corresponding frozen tumor sample pairs were analyzed for expression of the genes coding for cyclin D1 and p27, CCND1 and CDKN1B, respectively.

**Results:**

Forty-two patients completed all study parts, and immunohistochemical evaluation of ER and PR was achievable in 30 tumor pairs, HER2 in 29 tumor pairs, cyclin D1 in 30 tumor pairs and p27 in 33 tumor pairs. The expression of ER, PR and HER2 did not change significantly following atorvastatin treatment. Cyclin D1 expression in terms of nuclear intensity was significantly decreased (P = 0.008) after statin treatment in paired tumor samples. The protein expression of the tumor suppressor p27, evaluated either as the fraction of stained tumor cells or as cytoplasmic intensity, increased significantly (P = 0.03 and P = 0.02, respectively). At the transcriptional level, no significant differences in mRNA expression were detected for cyclin D1 (CCND1) and p27 (CDKN1B). However, CCND1 expression was lower in tumors responding to atorvastatin treatment with a decrease in proliferation although not significantly (P = 0.08).

**Conclusions:**

We have previously reported statin-induced anti-proliferative effects in breast cancer. This study suggests that cell cycle regulatory effects may contribute to these anti-proliferative effects via cyclin D1 and p27.

**Electronic supplementary material:**

The online version of this article (doi:10.1186/s12967-015-0486-0) contains supplementary material, which is available to authorized users.

## Background

**Figure 1:**
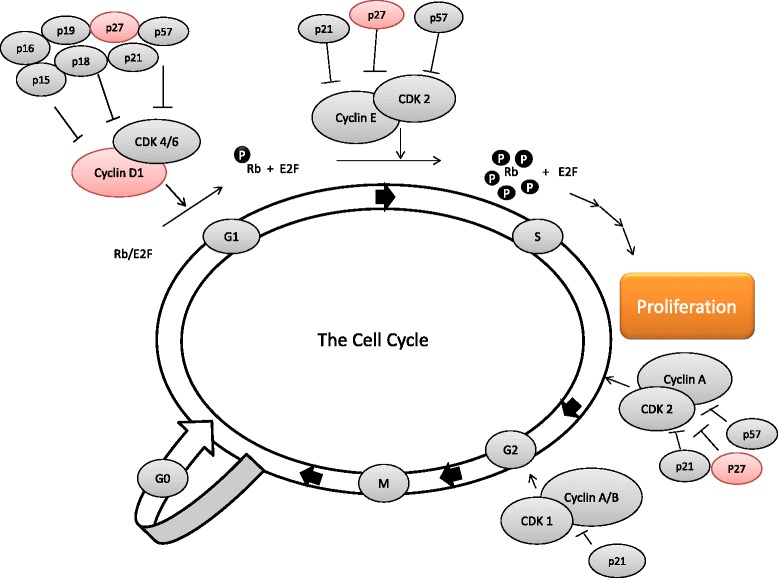
The cell cycle, and the main actions of cyclin D1 and p27. Cyclin D1 regulates the G1/S-phase transition, binds and activates Cdk4/Cdk6 to phosphorylate the retinoblastoma (pRb) protein. Phosphorylation of Rb leads to separation from E2F, and allows the transcription of proliferation genes [21]. In G0 and early G1, p27 inhibits CDK2-cyclin E, and in S-phase CDK2-cyclin A. In G1 there is a decrease in p27, allowing CDK2-cyclin E and CDK2-cyclin A to activate the transcription of genes acquired for the G1-S-transition [25]. P27 also interacts with CDK4/6-cyclin D comprehensively, p27 acting as both an inhibitor and as a required assembly factor for the complex, depending on the growth state of the cell [24].

Statins, a major class of drugs for treatment of hypercholesterolemia, are widely used due to a notable prevention of cardiovascular disease, and accumulating evidence proposes a promising role of statins in breast cancer [1]. Statins act by inhibiting 3-hydroxy-3methylglutaryl coenzyme-A reductase (HMGCR), the rate-limiting enzyme of the mevalonate pathway, thereby reducing intracellular cholesterol production [2]. In addition to their lipid-lowering capacity, statins exert several other effects mediated by different products of the mevalonate pathway. These lipid-independent effects include inhibition of inflammatory responses, immunomodulatory actions, apoptotic and anti-proliferative effects, which might contribute to the suggested anti-tumoral effects of these agents [3,4]. The epidemiological evidence projecting statins as anticancer agents is variable, depending on the particular type of cancer in question as well as the class of statin used [5-9]. Recent data suggest that lipophilic statins may be preferable over hydrophilic statins as anticancer agents [10,11]. In breast cancer, previous studies have shown lipophilic statin use following a breast cancer diagnosis to be associated with a decreased risk of disease recurrence and with reduced breast-cancer mortality [8,12,13]. Results from a phase II study with statins prescribed in the pre-surgical setting have demonstrated reduced tumor cell proliferation and increased apoptosis in patients with high grade *in situ* breast cancer [14]. The anti-proliferative effects of statins were confirmed in invasive breast cancer, as reported in a previous publication from the same trial on which this study is based [15]. In both studies, the anti-proliferative effects were described in terms of decreased intra-tumoral levels of Ki67 [14,15]. However, the comprehensive biological mechanisms behind this anti-proliferative effect are currently not clear. Ki67 is the most widely used clinical biomarker for assessing the proliferative status of a breast cancer. Ki67 is expressed during all active phases of the cell cycle (G1, S, G2, M), but is absent in resting cells (G0) [16,17]. The cell cycle is a complex and strictly controlled series of events, driving cell division and replication of DNA. In normal cells, progression through the cell cycle is controlled by the cyclin dependent kinases (CDKs), a family of serine/threonine kinases [18]. The CDKs form complexes with their regulatory units, cyclins, thereby activating the CDKs, leading to phosphorylation of the cell cycle regulatory proteins that initiate and regulate progression through the different phases of the cell cycle [19]. In breast cancer cells, the cell cycle control system is deregulated at multiple levels, leading to abnormal cell proliferation [20]. Cyclin D1 is a vital regulator of the G1/S transition, as illustrated in Figure 1. The interaction of cyclin D1 with CDK4 and CDK6, leads to phosphorylation and thereby inactivation of the Rb-protein and its G1-maintaining function, which culminates in the expression of proliferation-associated target genes [21,22]. Cyclin D1 is overexpressed at the protein level in up to 50% of all primary breast cancers, in part due to amplification of the cyclin D1 gene, CCND1 [23]. The CDK inhibitor p27, also known as Kip1, is involved in the regulation of the G0-to-S-phase transition. p27 interacts with CDK2-cyclin E, CDK4/6-cyclin D, and CDK2-cyclin A complexes, thereby regulating these complexes strictly [24,25]. The tumor suppressor p27 is frequently deregulated in breast cancer, and reduced p27 expression has been associated with increased proliferation, high tumor grade, HER2 amplification as well as estrogen receptor (ER) and progesterone receptor (PR) negativity [25,26].

The aim of this study was to investigate potential statin-induced effects on the central cell cycle regulators cyclin D1 and p27, to improve the understanding of the statin induced anti-proliferative effects previously reported. A secondary aim was to evaluate the expression of clinically established biomarkers, such as the estrogen receptor, progesterone receptor and HER2 before and after atorvastatin treatment, hypothesizing no statin-induced changes of their expression. These aims were addressed in a phase II window-of-opportunity trial with two-week, pre-operative high-dose atorvastatin therapy in 50 patients with primary invasive breast cancer.

## Materials and methods

### Trial design

The trial was designed as a window-of-opportunity study, in which the participants were prescribed the lipophilic statin atorvastatin for two weeks, during the treatment-free window between breast cancer diagnosis and surgery. The study was opened for recruitment in February 2009, and the pre-planned number of 50 patients was achieved in March 2012. In this non-randomized phase II trial, all patients received an equal dose of 80 mg atorvastatin daily. The trial was conducted as a single center study at Skåne University Hospital in Lund, Sweden.

The Ethical Committee at Lund University and the Swedish Medical Products Agency approved this trial. The study has been registered at ClinicalTrials.gov (i.e., ID number: NCT00816244, NIH). The study adheres to the REMARK criteria [27].

### Patients and tumors

Patients diagnosed with primary invasive breast cancer with a minimum tumor size of 15 mm measured by ultrasound, who were candidates for radical surgery, were eligible for participation in this study. A performance status below 2 according to the European Cooperative Oncology Group (ECOG) and normal liver function were also required for inclusion. Pregnancy, on-going hormonal replacement therapy, cholesterol lowering therapy (i.e., including statins, fibrates, and ezetemibe), a medical history of hemorrhagic stroke or allergic reactions attributed to compounds with a similar biological composition to that of atorvastatin encompassed the exclusion criteria. Complete information regarding the study inclusion and exclusion criteria, as well as clinical and pathological characteristics of the patients and tumors have been described in detail previously [15]. Following inclusion, the participants underwent study specific tumor core biopsies prior to statin treatment initiation with one core biopsy being formalin-fixed immediately and one being fresh frozen at −80°C. Subsequent to the two-weeks statin treatment, breast surgery was performed according to standard surgical procedures, and tumor tissue was retrieved from the primary tumor at the Department of Pathology at Skåne University Hospital, Lund, Sweden. Of the 50 patients enrolled in the study, a total of 42 patients completed all study parts. Two patients were excluded from the trial since date of surgery was pre-scheduled after enrollment. Two patients were excluded due to elevated serum levels of alanine aminotransferase. One patient was excluded since the diagnose of invasive breast cancer was questioned, one patient left the study due to nausea and dizziness and two patients left due to personal reasons.

### Endpoints and tumor evaluation

The primary endpoint of the clinical trial was statin-induced anti-proliferative tumor response measured by a decrease in Ki67 expression, as previously reported [15]. The purpose of this sub-study was to investigate potential effects of statin treatment on the expression of ER, PR, and HER2 as well as the expression of the cell cycle regulators cyclin D1 and p27.

### Immunohistochemistry

Formalin-fixed and paraffin-embedded tumor tissue from core biopsies and surgical samples were cut into 3 to 4 μm sections and transferred to glass slides (Menzel Super Frost Plus), dried at room temperature, and baked in a heated chamber for 2 hours at 60^○^C. De-paraffinization and antigen retrieval was performed using PT Link (Dako Denmark A/S) using a high pH buffer. Staining was performed in an Autostainer *Plus* (Dako) using a di-amino-benzidine (DAB) based visualization kit (K801021-2, Dako). Counterstaining was performed using Mayer’s hematoxylin with antibodies against ER (SP1, Thermo Scientific, diluted 1:200), PR (Dako M3569, diluted 1:200), HER2 (4B5, Ventan BenchMark Ultra, Ventana Medical Systems, Inc. Tucson, Arizona, R.U.), cyclin D1 (Dako M3635, diluted 1:40), and p27 (Dako M7203, diluted 1:100).

### Tumor biomarker assessment

ER and PR expression was evaluated as the fraction of stained nuclei, using a five-grade scale (i.e. 0-1%, 2-10%, 11-50%, 51-75% and >75% of stained cells). HER2 was evaluated using the HercepTest guidelines (DAKO, Carpinteria, CA) for scoring of HER2. No staining observed in <10% of the tumor cells was scored 0, faint staining observed in >10% of the tumor cells was scored 1+, weak to moderate staining in >10% of the tumor cells was scored 2+, and strong staining in >10% of the tumor cells was scored 3+, according to the guidelines. Assessment of cyclin D1 and p27 protein expression was evaluated by considering the fraction of stained nuclei, using a five-grade scale (i.e. 0-1%, 2-10%, 11-50%, 51-75% and >75% of stained cells), and nuclear intensity and cytoplasmic intensity, using a four-grade scale (i.e. negative, weak, moderate or strong) (Additional file 1: Figure S1 and Additional file 2: Figure S2). For Ki67 assessment, 400 tumor cells were evaluated and Ki67 expression recorded as the fraction of positive nuclei using a continuous scale from 0 to 100 [15].

### RNA extraction

Total RNA was extracted from fresh frozen tumor samples using the Allprep DNA/RNA mini kit (QIAGEN, Valencia, CA) in a QIAcube (Qiagen) according to the manufacturer's instructions. The RNA integrity was assessed on an Agilent 2100 Bioanalyzer (Agilent, Santa Clara, CA) and RNA quantification was performed using a NanoDrop ND-1000 (NanoDrop Products, Wilmington, DE). The samples were hybridized to Human HT-12 v4.0 Expression BeadChips (Illumina Inc, San Diego, CA) in two batches at the SCIBLU Genomics Center at Lund University, Sweden (www.lth.se/sciblu). The Illumina probes were re-annotated using the R package illuminaHumanv4.db [28]. The microarray study was conducted within another sub-study of the trial and comprehensive analyses of the data are subject of currently unpublished work. Thus, in this study only analyses concerning the expression of the probes representing cyclin D1 and p27 are reported herein.

### Statistical analysis

All assessed immunohistochemical tumor variables were measured on ordinal scales. Changes in ER, PR, HER2, cyclin D1 and p27 protein expression between pre- and post-atorvastatin treatment samples were evaluated using the Wilcoxon matched-pairs signed-rank test. Spearman’s rho was used as a measure of the correlation between change in cyclin D1 and Ki67, and p27 and Ki67, respectively. To test for subgroup differences, the Linear-by-linear association was used. All tests were two-sided and differences with P-values below 5% were considered significant. The software packages Stata version 12.1 (StataCorp LP, College Station, TX, 2012) and IBM SPSS Statistics Version 19, were used for the data analysis.

For microarray data analysis, all data were initially pre-processed and normalized using the Quantile Normalization method [29]. The GenomeStudio Software V2011.1 was used for the analyses. Probe sets with signal intensity below the median intensity of negative control signals in 80% of the samples were excluded. Replicate probe sets were merged by the median of signal intensity values. A Significance Analysis of Microarrays (SAM) analysis was performed using the TMeV v4.9 software to identify differences in expression of CCND1 and CDKN1B between paired pre- and post- statin treatment samples. Furthermore, changes in the expression of CCND1 and CDKN1B between tumor pairs stratified into two groups according to statin-induced changes in Ki67 expression were evaluated using the Mann–Whitney U-test. Changes in tumor proliferative rate, quantified by IHC analysis of the expression of Ki67, have been previously reported [15].

## Results

### Patient characteristics and tumor data

Fifty patients entered the trial; a total of 41 patients were reported as postmenopausal and nine patients as premenopausal. Forty-two patients completed all study parts. No serious adverse events were reported. At the time of diagnosis, the average age among all 42 patients was 63 years (range 35–89 years). The average pathological tumor size was 21 mm, ranging from 6 to 33 mm and all 42 tumors were invasive breast cancers. Most tumors were ductal cancers, and the majority of tumors were histological grade 2 or 3 (Table 1).

**Table 1:** **Patient- and tumor characteristics**

**Patients completed all study parts**	**n = 42**
Age years (mean, range)	63 (35–89)
Tumor size mm (mean, range)	21 (6–33)
Positive nodal status	17 (41%)
Tumor grade (NHG)	
I	9 (21%)
II	17 (41%)
III	16 (38%)
Mitotic index	
1	23 (55%)
2	5 (12%)
3	14 (33%)
ER (n = 30)	
Positive	27 (90%)
Negative	3 (10%)
PR (n = 30)	
Positive	24(80%)
Negative	6 (20%)
HER2 (n = 29)	
0	7 (24%)
1+	10 (34%)
2+	7 (24%)
3+	5 (17%)
Ki67 index (n = 26)	
Low	15 (58%)
High	11 (42%)
HMGCR (n = 38)	
Positive	24 (63%)
Negative	14 (37%)

NHG Nottingham histologic grade I-III (post-treatment pathological report), Mitotic index according to Nottingham criteria (post-treatment pathological report).

Baseline tumor data (pre-treatment): ER (estrogen receptor), PR (progesterone receptor), HER2 (human epidermal growth factor receptor 2), Ki67 high if >20%, HMGCR positive if any cytoplasmic staining.

### Changes in the expression of ER, PR, HER2

The evaluation of ER and PR was achievable in 30 tumor pairs and HER2 in 29 pairs, respectively, whereas the remaining pre-treatment biopsies showed insufficient amount of tumor tissue for immunohistochemical evaluation of these markers. The baseline expression of ER, PR and HER2 is shown in Table 1. When contrasting the pre-and post-treatment samples, neither ER, PR nor HER2 changed significantly (Wilcoxon matched-pairs signed-rank test P = 0.68, P = 0.19, and P = 0.08 for ER, PR and HER respectively; Table 2) and the null hypothesis of equal expression before and after statin treatment was retained.

**Table 2:** **Change in tumor expression from baseline (i.e. before atorvastatin treatment) to time of surgery (i.e. after atorvastatin treatment)**

	**Complete pairs**	**Decreasing**	**Unaltered**	**Increasing**	**P-value**
ER	*n =* 30	2	25	3	0.68
PR	*n =* 30	3	21	6	0.19
HER2	*n =* 29	7	20	2	0.08
Cyclin D1 nuclear fraction	*n =* 30	4	19	7	0.12
Cyclin D1 nuclear intensity	*n =* 30	14	13	3	0.008*
Cyclin D1 cytoplasmic intensity	*n =* 30	10	14	6	0.48
p27 nuclear fraction	*n =* 33	2	22	9	0.03
p27 nuclear intensity	*n =* 33	9	18	6	0.35
p27 cytoplasmic intensity	*n =* 33	3	18	12	0.02

P-values from Wilcoxon matched-pairs signed-ranks test.

ER estrogen receptor, PR progesterone receptor, HER2 human epidermal growth factor receptor 2.

*Significant even after Bonferroni adjustment for multiple testing within the marker, P = 0.02.

### Changes in the expression of cyclin D1

**Figure 2:**
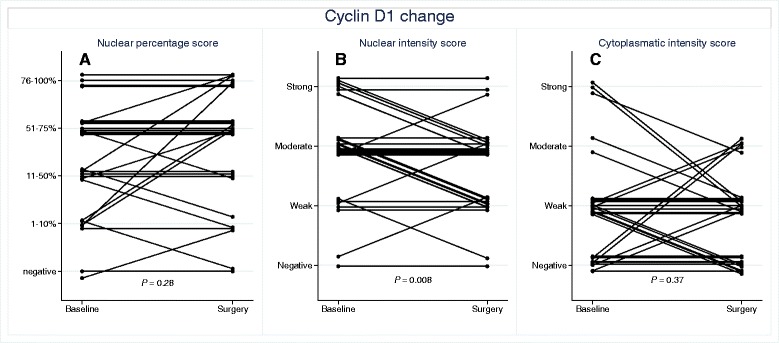
Change in tumor expression of cyclin D1 from baseline (i.e., before atorvastatin treatment) to time of surgery (i.e., after atorvastatin treatment). **A)** Fraction of stained nuclei; **B)** Nuclear intensity; **C)** Cytoplasmic intensity. To reduce the problem of completely overlapping lines in the spaghetti plot, for each pair of pre/post-treatment samples, a random number from a uniform distribution over the interval [−0.15, 0.15] was added, shifting the corresponding line at most 15%, upwards or downwards, of a step on the integer-valued score scale.

Immunohistochemical evaluation of cyclin D1 was achievable in 30 of the 42 paired samples restricted by insufficient amount of tumor tissue in the remaining core biopsies. Table 3 shows cyclin D1 expression in the pre-treatment samples. A comparison of the expression of cyclin D1 between pre-and post-treatment samples is shown in Figure 2. In general, the majority of samples expressed cyclin D1. However, the nuclear intensity of the protein expression was significantly decreased (P = 0.008, Wilcoxon matched-pairs signed-rank test) following statin treatment (Table 2). Furthermore, cyclin D1 expression was assessed regarding the fraction of stained nuclei as well as the intensity of cytoplasmic staining, but neither the nuclear fraction nor the cytoplasmic intensity changed significantly following treatment. No significant association was found between the pre-treatment tumor characteristics in relation to the change in cyclin D1 following atorvastatin treatment (Additional file 3: Table S1).

**Table 3:** **Cyclin D1 and p27 tumor expression in the pre-treatment setting**

**Patients completed all study parts**	**n = 42**
Cyclin D1 nuclear fraction (*n* = 30)	
Negative	2 (7%)
Low (1-50%)	12 (40%)
High (51-100%)	16 (53%)
Cyclin D1 nuclear intensity (*n* = 30)	
Negative	2 (7%)
Weak	5 (12%)
Moderate	14 (33%)
Strong	9 (30%)
Cyclin D1 cytoplasmic intensity (*n* = 30)	
Negative	11(37%)
Weak	14 (47%)
Moderate	2 (7%)
Strong	3 (10%)
p27 nuclear fraction (*n* = 33)	
Negative	0
Low (1-50%)	8 (24%)
High (51-100%)	25 (76%)
p27 nuclear intensity (*n* = 33)	
Negative	0
Weak	3 (9%)
Moderate	18 (55%)
Strong	12 (36%)
p27 cytoplasmic intensity (*n* = 33)	
Negative	17 (52%)
Weak	14 (42%)
Moderate	2 (6%)
Strong	0

### Changes in the expression of p27

**Figure 3:**
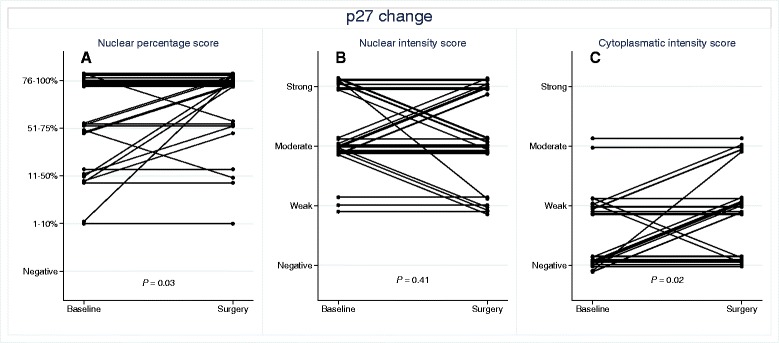
Change in tumor expression of p27 from baseline (i.e., before atorvastatin treatment) to time of surgery (i.e., after atorvastatin treatment). **A)** Fraction of stained nuclei; **B)** Nuclear intensity; **C)** Cytoplasmic intensity. To reduce the problem of completely overlapping lines in the spaghetti plot, for each pair of pre/post-treatment samples, a random number from a uniform distribution over the interval [−0.15, 0.15] was added, shifting the corresponding line at most 15%, upwards or downwards, of a step on the integer-valued score scale.

Immunohistochemical evaluation of p27 could be performed for 33 of the 42 paired tumor samples. Prior to atorvastatin treatment, all samples demonstrated tumor cells expressing p27 to a different extent as shown in Table 3. Following atorvastatin treatment there was a significant increase in the fraction of tumor cells expressing p27 (P = 0.03, Wilcoxon matched-pairs signed-rank test, Table 2 and Figure 3). The nuclear intensity of p27 did not change significantly (P = 0.35). Further, the cytoplasmic intensity of p27 was significantly increased after atorvastatin treatment (P = 0.02, Wilcoxon matched-pairs signed-rank test). Baseline tumor characteristics in relation to the change in p27 expression following atorvastatin treatment are summarized in Additional file 4: Table S2, for which no significant associations were found.

### Correlation between change in Ki67 and change in cyclin D1 or p27

**Figure 4:**
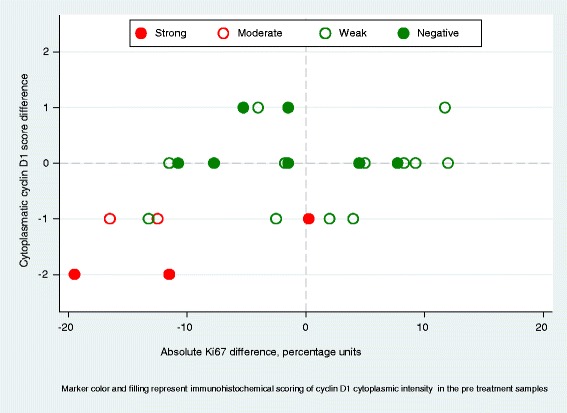
Correlation between change in Ki67 and change in cyclin D1 (cytoplasmic intensity) after treatment with atorvastatin. Marker color and filling represents immunohistochemical scoring of cyclin D1 cytoplasmic intensity in the pre-treatment samples; filled red circles (strong cyclin D1 intensity), red empty circles (moderate cyclin D1 internsity), green empty circles (week cyclin D1 intensity), green filled circles (no cyclin D1 expression).

Spearman´s correlation was used to evaluate whether a change in the expression of cyclin D1 or p27 was accompanied by a change in proliferation as determined by Ki67. We observed that a decrease in Ki67 corresponded positively with a decrease in cytoplasmic intensity of cyclin D1 (N = 25, P = 0.03, Spearman’s rho = 0.43), as illustrated in Figure 4. No significant associations were detected between the decrease in Ki67 and the change in nuclear fraction or nuclear intensity of cyclin D1, or the change in Ki67 and the change in p27 irrespective of cellular localization or staining intensity.

### mRNA expression of proliferation associated genes

**Figure 5:**
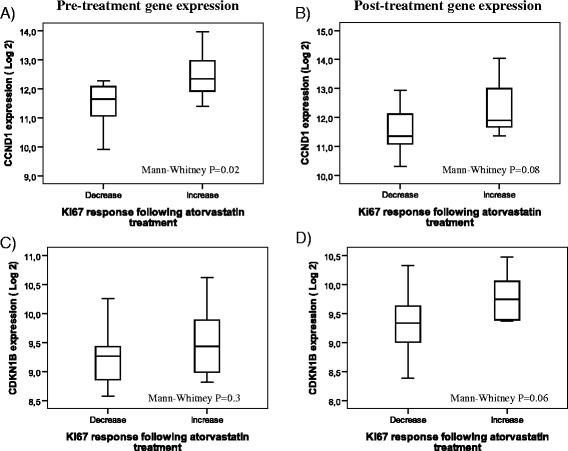
Expression of CCND1 and CDKN1B pre- and post-atorvastatin treatment, divided into tumors responding with a decrease or increase in proliferation (Ki67) following statin treatment. **A)** Pre-treatment CCND1 expression, **B)** Post-treatment CCND1 expression, **C)** Pre-treatment CDKN1B expression, **D)** Post-treatment CDKN1B expression.

Initially, we compared the expression of CCND1 and CDKN1B between paired pre-and post-treatment samples. Good quality gene expression data were available for twenty-five tumor pairs; no statistically significant difference in the expression of these genes was noted. Next, a sub-analysis comparing the mRNA levels of CCND1 and CDKN1B was performed after dividing samples into two groups based on changes in Ki67 expression as assessed by IHC. Ki67 expression was decreased in 15 samples while 10 samples showed an increased expression as previously reported [15]. Separate analyses were performed for the pre- and post-treatment samples. As illustrated in Figure 5A, the expression of CCND1 in the pre-treatment samples was significantly correlated to response in tumor cell proliferation (P = 0.02; Mann–Whitney). Correspondingly, in the post-treatment samples, a marginally lower CCND1 expression was observed among the tumors responding with a decrease in Ki67 compared to tumors with an increase in Ki67 (Figure 5B; P = 0.08; Mann–Whitney). CDKN1B mRNA expression did not differ significantly between tumors responding with a Ki67 response or not (Figure 5C-D; P = 0.3, 0.06: Mann–Whitney).

## Discussion

In the present study, we investigated the effects of short-term administration of a high-dose of atorvastatin on the conventional breast cancer pathological markers ER, PR, HER2, as well as the cell cycle regulators cyclin D1 and p27. Our results indicate that ER, PR and HER2 expression remain stable following treatment with atorvastatin. However, a significant decrease in cyclin D1 expression and a significant increase in p27 expression were observed, indicating that the anti-proliferative effects of statins may be driven by the cell cycle regulatory effects of cyclin D1 and p27.

There is a rising interest in statins, due to their effects extending beyond their well-known lipid-lowering capacity [3]. As previously reported from this trial, a significant decrease in tumor proliferation, in terms of decrease in Ki67 expression, was noted especially in the sub-set of tumors expressing HMGCR at baseline [15]. This difference in proliferation may be driven by changes in the cell cycle regulators cyclin D1 and p27, as has been addressed in this study. It has been proposed that the anti-proliferative and pro-apoptotic effects of statins are due to the inhibition of downstream isoprenoid intermediates, such as farnesyl-pyrophosphate (FPP) and geranyl-geranyl-pyrophosphate (GGPP) [30-32]. FPP and GGPP are molecules which post-translationally modify a number of proteins by creating a hydrophobic domain, thereby allowing the proteins to anchor to cell membranes and perform their normal functions, a process known as protein prenylation [33]. Protein prenylation is necessary for the activation of many proteins participating in signaling pathways on which tumors depend, such as the RAS/Rho superfamily. RAS-dependent pathways regulate the expression of both p27 and cyclin D1, the assembly of cyclin D1 with CDK4/6, and the growth factor-induced regulation, transcription, and stabilization of cyclin D1 [34].

In concordance with our results, statins have been shown to inhibit cell growth, with G1 arrest, leading to reduced transition to the S and G2/M phases of the cell cycle [35]. Both cyclin D1 and p27 are involved in the regulation of these transitions, cyclin D1 through the association with CDK4 and CDK6, and p27 by interacting with the CDK2/cyclin E, CDK2/cyclin A, and CDK4/6-cyclin D complexes. A decrease in cyclin D1 entails that p27 is released from the CDK4/6-cyclin D complex, and instead able to assemble with, and inhibit CDK2, thereby promoting cell cycle arrest and inhibit proliferation [24]. This suggests that a statin induced cell cycle arrest at G1 could be the result from a decrease in cyclin D1 expression, and a corresponding increase in p27 as suggested by our data. Previous *in vitro* studies have shown similar results with a statin induced up-regulation of p27 [36-38] and reduced levels of cyclin D1 [39] in various tumor cell lines. Cyclin D1 and p27 are both regulated by a plethora of different signal transduction pathways [25,40], and the underlying mechanisms of the observed decrease of cyclin D1 and increase of p27 in this study is not evident. Given the suggested effects of statins on cell cycle regulators and the recent approval of a CDK4/6-inhibitor for first-line treatment of advanced ER positive breast cancer [41], further studies examining the potential synergistic effects of statins and CDK4/6 inhibitors would be of clinical importance.

The expression of clinically established biomarkers such as ER, PR and HER2 was evaluated in both pre-and post-treatment samples to establish whether these markers were affected by statin treatment. The vast majority of samples pairs remained unchanged. Recently, the cholesterol metabolite 27-hydroxycholesterol (27HC), has been revealed to increase ER-dependent growth in mouse models of breast cancer [42]. In the endocrinological field of research, 27HC has been linked to a decrease in bone mineral density, in part due to its ability to bind to and modulate the transcriptional activity of ER [43]. An *in vitro* study showed that simvastatin exerted osteoinductive effects, partly achieved through an increase in ER expression [44]. Regarding HER2, signaling through this receptor is dependent on the cholesterol content of the lipid rafts [45]. Thus, statins may theoretically enable changes in the expression of both steroid receptors and HER2. Such changes were not detected in this study. However, the treatment duration of only two weeks might be insufficient to índuce changes in ER or PR expression, due to their relatively stable nature [46,47]. The absence of a significant change in the expression of ER, PR and HER2 might be of clinical interest, indicating that statin treatment can be administrated safely to breast cancer patients without altering clinically used prognostic and treatment predictive markers. In the immunohistochemical evaluations of cyclin D1 and p27, expression was scored for both percentage of positive nuclei, nuclear intensity and cytoplasmic intensity. Currently, established scoring systems for immunohistochemical evaluation of cyclin D1 and p27 are not available. In a review by Chu *et al.*, [25] most prognostic studies scored p27 based on the percentage of positive tumor nuclei, with various cut-offs. Others scored both the percentage of positive nuclei and the intensity of the staining. Most studies however omitted scoring of cytoplasmic expression of p27. In this study, immunohistochemical evaluations demonstrated significant changes regarding the cyclin D1 nuclear intensity, fraction of p27 stained cells, and the cytoplasmic intensity of p27. Both cyclin D1 and p27 exert their effects on the G1/S transition control when localized to the nucleus [25,48] A decrease of cyclin D1, results in p27 no longer being sequestrated by the CDK4/6-cyclin D complex to the same extent. Data suggest that the cell favours maintainance of low levels of p27 in the nuclear space, and subsequently mislocalize p27 to the cytoplasmic compartment when levels of nuclear p27 are increased [24], which may explain the concurrent increase in expression of p27 in both the nuclear and cytoplasmic compartments in this study. Importantly, data from functional studies suggest that cytoplasmic translocation of p27 can change its function in tumor cells [49], thus promoting other functions opposite to its tumor suppressor role, e.g. cell migration [50]. A review by Guan *et al.* concluded that further studies were needed to understand the role of cytoplasmic p27 in breast cancer [26]. However, the significance of the cellular localization of p27 cannot be explained based on the results from this study. The cytoplasmic intensity of cyclin D1 was associated with Ki67 (Figure 4), although expression did not change significantly during treatment. During G1, cyclin D1 accumulates in the nucleus, but is exported to the cytoplasmic space when the cell enters S-phase [48], possibly implying a more intense cytoplasmic cyclin D1 staining in high proliferating aggressive tumors, a correlation found in pancreatic adenocarcinoma [51], and suggestively explaining the positive correlation with Ki67. Further, gene expression analyses of paired tumor samples were performed. Only marginal changes in CCDN1 and CDKN1B expression were observed following two weeks of statin treatment. However, the cell cycle-dependent changes in cyclin D1 and p27 can both ensue through other mechanisms, including post transcriptional deregulation. [23,25]. The gene expression of CCND1 was found to be significantly correlated to response in tumor cell proliferation, indicating a difference in the response to statins between cancers with or without CCND1 overexpression.

Whether and how the dose or duration of statin treatment influences the here presented results is unclear and cannot be further elucidated from this trial, as all patients in the study were given atorvastatin for two weeks at the maximum recommended dose to optimize the drug delivery into the breast cancer cells. No serious adverse events were observed, and only one patient withdrew from the study due to side effects, indicating that the treatment with high-dose atorvastatin was well-tolerated during the two-week administration. To gain more insight regarding the statin-induced effects on expression of cell cycle regulators, prolonged treatment duration may be neccessary to demonstrate the maximal effect on cell cycle regulators. However, due to ethical considerations, this window-study was not able to extend the time from diagnosis to surgery, which restricted the duration of statin treatment to two weeks. Thus, as implied in the trial design and purpose of window-trials, these trials can generate adequate hypotheses which should preferably be evaluated in larger phase III trials [52]. As recently proposed by Ahern et al., the existing evidence supporting a protective effect of statins on breast cancer prognosis, is considered sufficient to launch a clinical phase III trial with statins in the adjuvant setting [1].

## Conclusions

In conclusion, the results from this window-of-opportunity study indicate a statin induced effect on central cell cycle regulators, in terms of an up-regulated expression of the tumor suppressor p27 and down-regulated expression of the oncogene cyclin D1 in breast cancer. The results are concordant with previous trial results, and suggest that cell cycle regulatory effects may be contributing to the anti-proliferative effects via cyclin D1 and p27.

## Additional files

Additional file 1: Figure S1. Examples of immunohistochemical cyclin D1 staining with negative nuclear and cytoplasmic expression **(a)**, weak nuclear and cytoplasmic expression **(b)**, moderate nuclear and cytoplasmic expression **(c)**, and strong nuclear and weak cytoplasmic expresssion **(d)**, respectively. Additional file 2: Figure S2. Examples of immunohistochemical p27 staining with weak nuclear and negative cytoplasmic **(a)**, moderate nuclear and weak cytoplasmic **(b)**, moderate nuclear and cytoplasmic **(c)**, and strong nuclear and moderate cytoplasmic **(d)** expression, respectively. Additional file 3: Table S1. Core biopsy tumor characteristics in relation to the change in cyclin D1 nuclear fraction, nuclear intensity and cytoplasmic intensity, pre- to post-atorvastatin. Additional file 4: Table S2. Core biopsy tumor characteristics in relation to the change in p27 nuclear fraction, nuclear intensity and cytoplasmic intensity, pre- to post-atorvastatin.
