# Supplementary material for: Statin-induced anti-proliferative effects via cyclin D1 and p27 in a window-of-opportunity breast cancer trial
Source: J Transl Med. 2015 Apr 29;13:133. doi: 10.1186/s12967-015-0486-0 (PMC4424530; doi:10.1186/s12967-015-0486-0)
Supplement: Additional file 1: Figure S1. — Examples of immunohistochemical cyclin D1 staining with negative nuclear and cytoplasmic expression (a), weak nuclear and cytoplasmic expression (b), moderate nuclear and cytoplasmic expression (c), and strong nuclear and weak cytoplasmic expresssion (d), respectively. [file 12967_2015_486_MOESM1_ESM.pdf]

Supplementary Figure S1

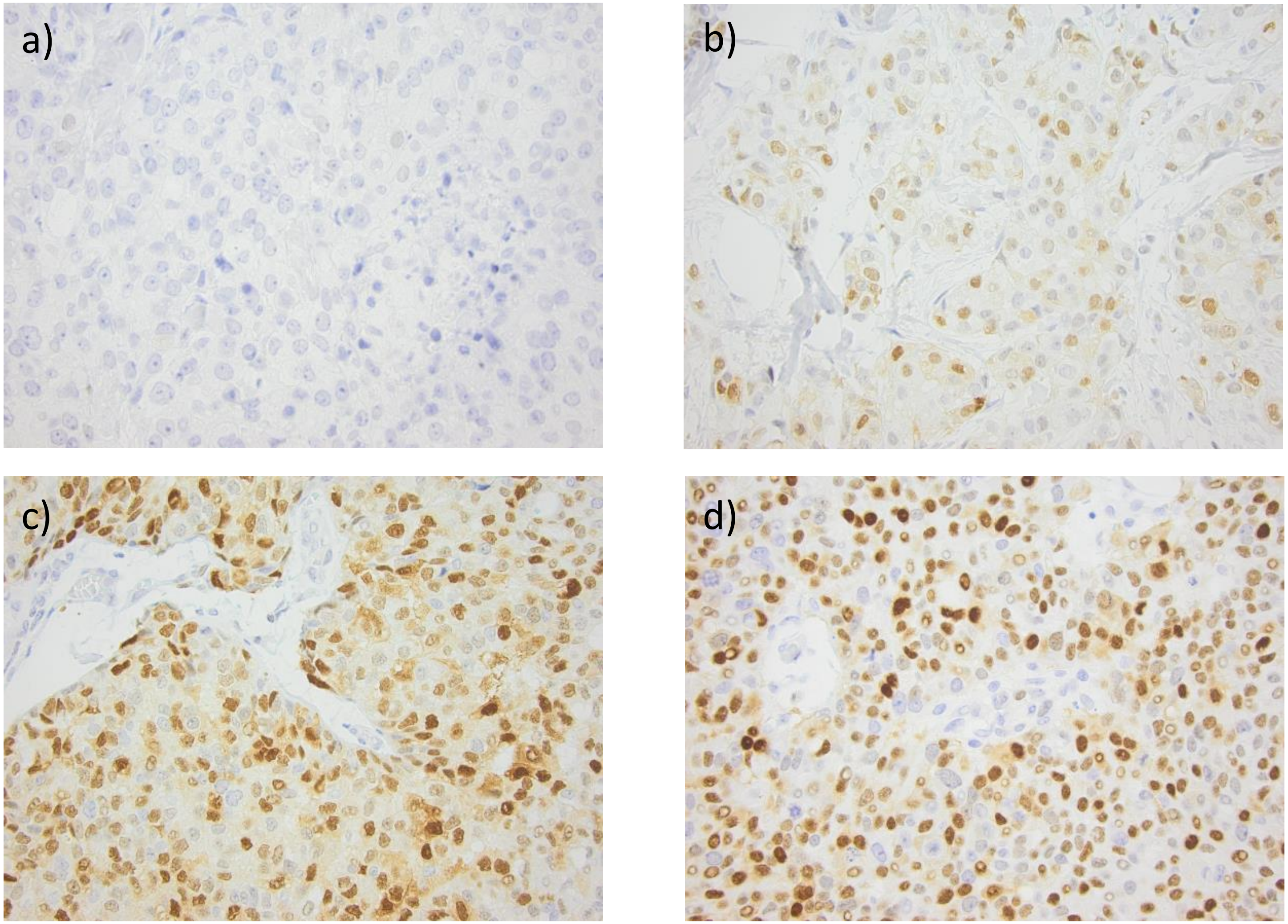

Supplementary Figure 1. Examples of immunohistochemical cyclin D1 staining with negative nuclear and cytoplasmic expression (a), weak nuclear and cytoplasmic expression (b), moderate nuclear and cytoplasmic expression (c), and strong nuclear and weak cytoplasmic expresssion (d), respectively.
