# Supplementary material for: Statin-induced anti-proliferative effects via cyclin D1 and p27 in a window-of-opportunity breast cancer trial
Source: J Transl Med. 2015 Apr 29;13:133. doi: 10.1186/s12967-015-0486-0 (PMC4424530; doi:10.1186/s12967-015-0486-0)
Supplement: Additional file 2: Figure S2. — Examples of immunohistochemical p27 staining with weak nuclear and negative cytoplasmic (a), moderate nuclear and weak cytoplasmic (b), moderate nuclear and cytoplasmic (c), and strong nuclear and moderate cytoplasmic (d) expression, respectively. [file 12967_2015_486_MOESM2_ESM.pdf]

Supplementary Figure S2

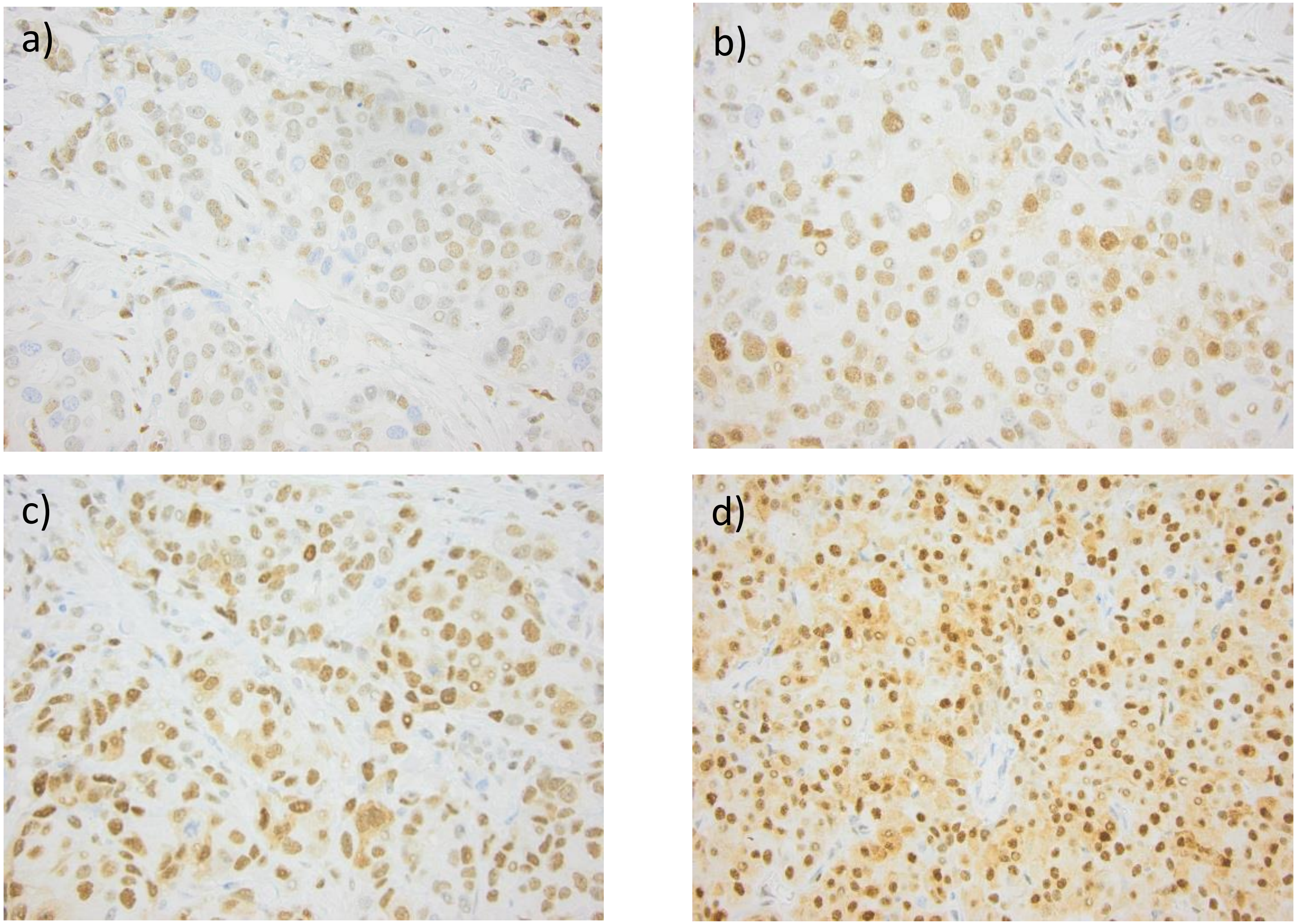

Examples of immunohistochemical p27 staining with weak nuclear and negative cytoplasmic (a), moderate nuclear and weak cytoplasmic (b), moderate nuclear and cytoplasmic (c), and strong nuclear and moderate cytoplasmic (d) expression, respectively.
